# Fatal Leguminous Aspiration Diagnosed at Postmortem Examination: A Rare Cause of Sudden Death in a Previously Healthy Adult

**DOI:** 10.7759/cureus.113566

**Published:** 2026-07-29

**Authors:** Shinjini Choudhury, Kavita Gaur, Kiran Agarwal, Rahul Singh, Shobhakant S Singh

**Affiliations:** 1 Pathology, Lady Hardinge Medical College, New Delhi, IND

**Keywords:** aspiration pneumonitis, autopsy, foreign body aspiration, forensic pathology, histopathology, lentil aspiration, sudden death

## Abstract

Aspiration pneumonitis due to vegetative foreign material is a rare and underrecognized cause of sudden death, particularly in previously healthy individuals without conventional predisposing risk factors. We report the case of a 52-year-old man who was found unconscious at home and declared dead on arrival at the emergency department. Organs were received for medicolegal histopathological evaluation following external examination at the scene. Gross examination of the lungs revealed firm parenchyma with patchy consolidation and smooth, glistening pleural surfaces. Histopathological examination demonstrated alveolar edema, hemorrhage, and acute inflammatory infiltrates. The key diagnostic finding was the presence of multiple round-to-oval structures within a bronchiolar lumen, with lobulated outlines, optically empty centers, and a periodic acid-Schiff-positive outer coating consistent with leguminous particles, most likely lentil fragments. The particles were surrounded by foamy macrophages, supporting antemortem aspiration. Ziehl-Neelsen staining was negative for acid-fast bacilli, and no granulomatous inflammation was identified. The cause of death was certified as aspiration pneumonitis due to lentil aspiration. This case highlights the need for meticulous histopathological examination of the airways during medicolegal evaluation, as food-related aspiration remains an underappreciated cause of sudden death in forensic settings where traditional risk factors are absent.

## Introduction

Aspiration pneumonitis is an inflammatory lung injury caused by the entry of gastric contents or foreign material into the lower respiratory tract. It is most commonly encountered in elderly, debilitated, or immunocompromised individuals and in patients with neuromuscular disorders predisposing them to dysphagia and, subsequently, the possibility of aspiration. However, it may also occur in apparently healthy adults under unrecognized circumstances [1,2].

Food-related aspiration is a well-documented cause of morbidity and mortality, with commonly implicated materials including meat, vegetables, nuts, grains, and bread [1,2]. Despite this, aspiration as a certified cause of death remains relatively uncommon, accounting for approximately 1%-3% of asphyxial fatalities, and is frequently underdiagnosed in forensic practice when the event is unwitnessed and gross findings are subtle [1,2].

Lentil aspiration produces two pathologically distinct patterns of pulmonary injury, determined by the tempo and volume of aspiration rather than by the aspirated material itself. Chronic microaspiration, recurrent low-volume inhalation over weeks to months, as seen in infants subjected to forceful feeding or in patients with longstanding neuromuscular dysphagia, allows the lung time to mount an organized immune response, producing hypersensitivity pneumonitis or foreign-body granulomatous reaction with multinucleated giant cells [3-5]. Acute macroaspiration, by contrast, involves sudden inhalation of a larger bolus causing immediate bronchiolar impaction, alveolar edema, hemorrhage, and an acute inflammatory exudate; granuloma formation does not occur because the patient does not survive long enough for an organized immune response to develop. All prior published cases were diagnosed in living patients through clinical evaluation, bronchoscopy, or surgical lung biopsy and represent the chronic microaspiration pattern [3-6].

One forensic case reported lentil-derived particles at autopsy in a child who died from traumatic hemorrhage; however, the particles were attributed to perimortem gastric contamination rather than antemortem aspiration, and lentil aspiration was not the cause of death [7]. To the best of our knowledge, no prior publication has documented a medicolegal case in which aspiration of leguminous material was certified as the direct and sole cause of death in a previously healthy adult, presenting as an acute macroaspiration pneumonitis without preceding clinical suspicion. We present this case to highlight leguminous aspiration as a potentially overlooked cause of sudden death in forensic settings and to describe reproducible histopathological and histochemical criteria for distinguishing antemortem aspiration from agonal or postmortem gastric contamination.

## Case presentation

As this was a medicolegal autopsy performed under statutory authority, informed consent was not required. The report has been anonymized to protect patient identity.

A 52-year-old man was found unconscious at home by family members and was declared dead on arrival at the emergency department. There was no documented history of trauma, poisoning, or significant pre-existing illness. No history of dysphagia, neurological disease, or prior respiratory illness was available from the scene investigation.

External examination of the body was performed at the scene by the investigating authority. The body was subsequently transferred, and organs were received at our institution for medicolegal histopathological evaluation. No injuries suggestive of trauma were identified on external examination. The received organs showed no gross evidence of internal hemorrhage, and no gross traumatic cause of death was identified. Both lungs were firm and showed patchy areas of consolidation. The pleural surfaces were smooth and glistening. Other received organs were grossly unremarkable. Toxicological analysis was negative for ethanol, opiates, benzodiazepines, organophosphates, and other agents included in the standard forensic toxicology panel. There was no toxicological evidence of poisoning as a contributory cause of death.

**Figure 1:**
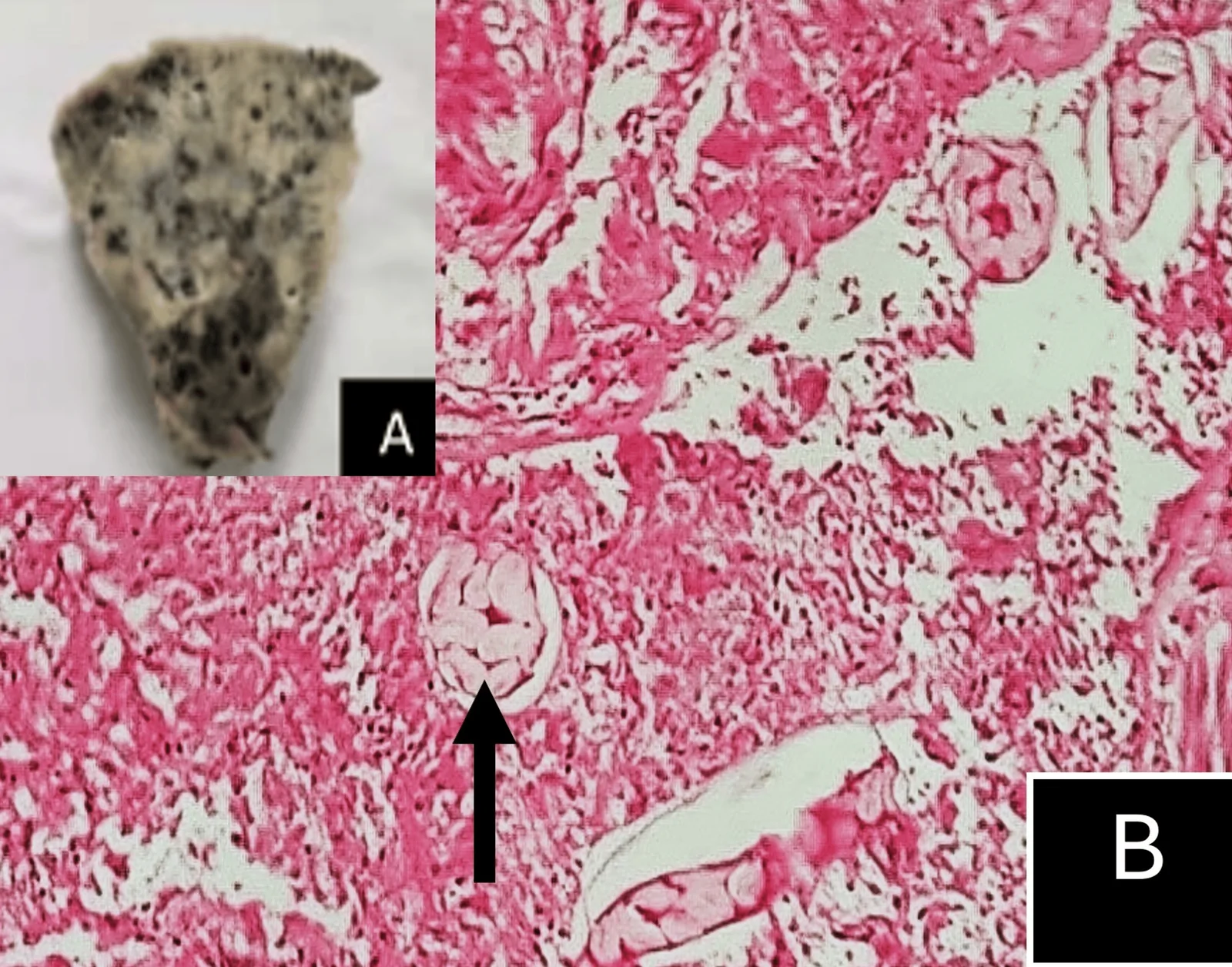
Gross and hematoxylin and eosin findings in fatal lentil aspiration. Panel A shows the gross lung specimen with consolidation and loss of normal sponginess. Panel B shows a round-to-oval aspirated structure within the airway lumen, with a lobulated outline, optically empty interior, and eosinophilic membranous coating, suggestive of leguminous material (hematoxylin and eosin, 200x).

**Figure 2:**
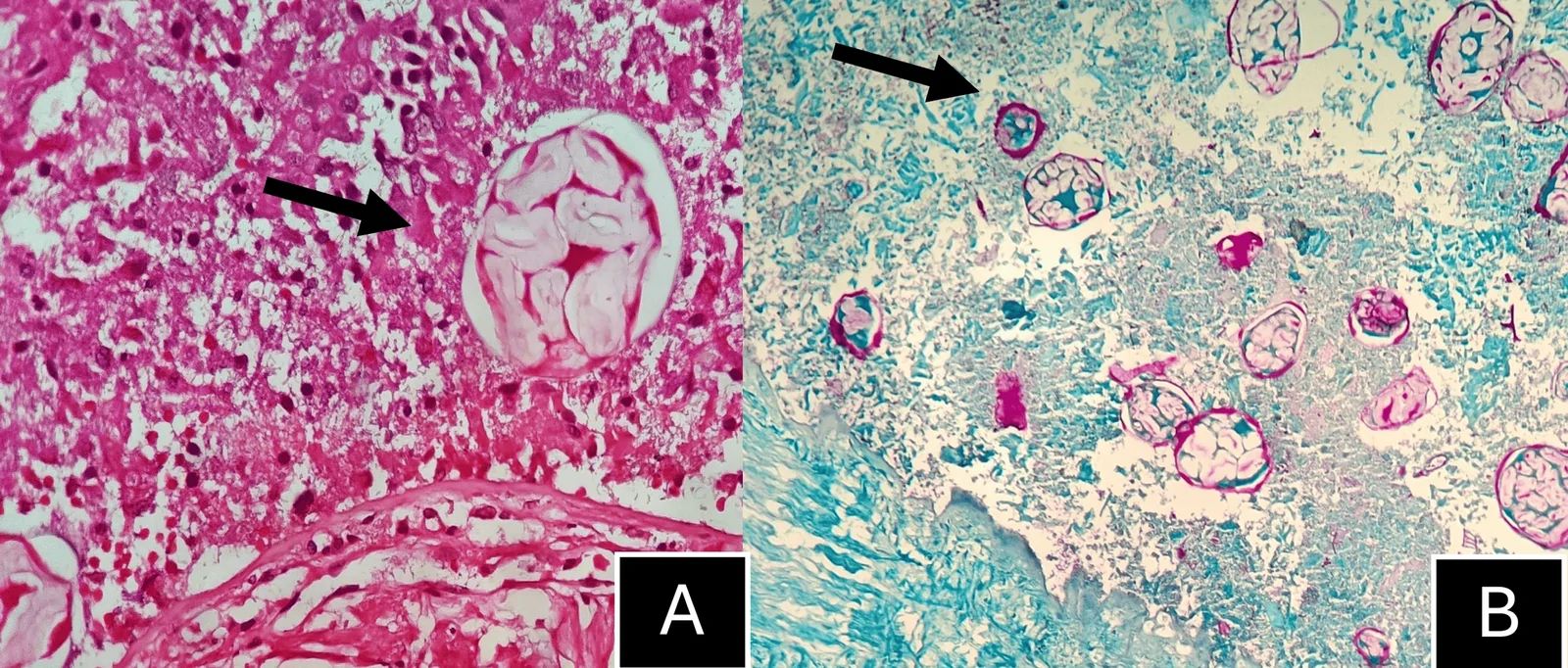
Higher-power morphology and histochemical confirmation of aspirated leguminous material Panel A shows a round-to-oval structure with lobulated, optically empty interiors and an eosinophilic membranous coating, suggestive of a lentil fragment (hematoxylin and eosin, 400x). Panel B shows strong periodic acid-Schiff positivity of the outer coating, reflecting the carbohydrate-rich composition of the plant-derived material (periodic acid-Schiff stain, 100x).

Histopathological examination of the lungs revealed alveolar spaces filled with edema fluid and hemorrhage, along with acute inflammatory infiltrates. Focal emphysematous changes and alveolar wall destruction were also noted. The key diagnostic finding was identified within a bronchiolar lumen, where multiple round-to-oval structures were seen. On hematoxylin and eosin staining, the particles appeared as discrete, non-birefringent bodies with a well-defined eosinophilic outer membrane and an optically clear interior. These structures showed lobulated outlines and were embedded within a mixed inflammatory exudate (Figures 1A-1B). The periodic acid-Schiff (PAS) positivity of the outer coating reflected the high polysaccharide content of the lentil seed coat (Figures 2A-2B). The particles were surrounded by foamy macrophages, supporting antemortem rather than agonal or postmortem aspiration of the material.

The morphological features are consistent with leguminous seed storage cells as described by Rosen et al. [7]. It should be noted that the microscopic appearances of seed storage cells derived from different leguminous species are virtually identical on routine light microscopy. The identification as lentil-derived is therefore presumptive, based on regional dietary prevalence, and definitive botanical speciation was not performed.

Ziehl-Neelsen staining was negative for acid-fast bacilli, and no granulomatous inflammation or fungal elements were identified. The kidneys showed tubular epithelial changes consistent with hypoxic-ischemic acute tubular necrosis; however, early postmortem autolysis producing morphologically similar appearances cannot be excluded, and no reliance is placed on the renal findings in determining the cause of death. Other organs were histologically unremarkable. Based on the cumulative findings, the cause of death was certified as aspiration pneumonitis due to aspiration of leguminous material, presumptively lentil.

## Discussion

Aspiration spans a spectrum of pulmonary injury ranging from mild chemical pneumonitis to severe airway compromise and death. In forensic practice, aspiration-related deaths can be underdiagnosed, particularly when the event is unwitnessed and gross findings are subtle or nonspecific [1,2]. This case documents an unusual aspiration-related death due to vegetative material, presumptively lentil fragments based on regional dietary prevalence, and reinforces that fatal food aspiration should remain in the differential diagnosis even in adults without classic predisposing conditions.

A key pathological distinction in leguminous aspiration is between chronic microaspiration and acute macroaspiration. Chronic microaspiration, recurrent low-volume inhalation over weeks to months in patients with predisposing neuromuscular or developmental conditions, allows the lung time to mount an organized foreign-body granulomatous response with multinucleated giant cells, as documented in prior published cases [3-5]. Acute macroaspiration, as in the present case, produces immediate bronchiolar impaction, alveolar edema, hemorrhage, and an acute inflammatory exudate without granuloma formation, as the patient does not survive long enough for an organized immune response to develop. The differing histological pattern between the Ros case and the present case therefore reflects the tempo of aspiration rather than a difference in the aspirated material [3].

Mukhopadhyay and Katzenstein demonstrated that particulate matter aspiration is more prevalent than clinically recognized and may mimic a range of pulmonary conditions; they emphasized that diagnosis depends on identifying foreign material in conjunction with the surrounding tissue reaction [8]. In this case, the histological pattern was that of acute aspiration pneumonitis with alveolar edema, hemorrhage, and acute inflammatory infiltrates combined with bronchiolar impaction by vegetative leguminous material. The aspirated particles displayed round-to-oval forms with lobulated outlines, optically empty centers, and a PAS-positive outer coating reflecting their high plant polysaccharide content. Rosen et al. demonstrated that these morphological features are characteristic of seed storage cells derived from leguminous and non-leguminous plants, and that the species of origin cannot be determined by routine light microscopy alone [7]. The identification as lentil-derived is therefore presumptive, based on regional dietary prevalence, and definitive botanical speciation was not performed. Surrounding foamy macrophages provided additional forensic weight, supporting antemortem aspiration rather than passive agonal or postmortem contamination of the tracheobronchial tree [6,8].

Leguminous plants contain lectins (phytohemagglutinins) that can directly injure bronchiolar epithelium and activate macrophages independently of mechanical obstruction [9]. In undercooked legumes, lectin content may be substantially higher than in fully processed material, potentially amplifying the severity of the acute pneumonitic response beyond what airway impaction alone would produce.

The main histopathological differentials when foreign particulate material is suspected include hypersensitivity pneumonitis, sarcoidosis (distinguished by non-caseating epithelioid granulomas), tuberculosis (distinguished by acid-fast bacilli positivity on Ziehl-Neelsen staining), and metastatic malignancy. In this case, Ziehl-Neelsen staining was negative for acid-fast bacilli, and no granulomatous inflammation was identified, effectively excluding the principal infectious and granulomatous mimics. Careful airway sampling and correlation of particle morphology with the inflammatory response are central to establishing the diagnosis; prior clinicopathologic work supports using the combination of foreign material and an appropriate host response as the most defensible diagnostic criterion [8,10].

Although food-related aspiration represents a small fraction of certified asphyxial deaths, it is potentially preventable [1,2]. Preventive measures include swallowing assessment, dietary texture modification in at-risk populations, and caregiver education regarding choking hazards. In the forensic setting, a practical recommendation is to document and preserve any suspected food material at the scene, including vomitus, food remnants, or nearby objects, for correlation with histologic particle morphology and staining results, while maintaining strict attention to contamination risk during tissue sampling [6]. This case highlights the value of meticulous airway examination and targeted histopathological sampling in all cases of sudden or unexplained death, where failure to recognize aspirated organic material may lead to misclassification of the cause of death.

## Conclusions

Fatal aspiration of leguminous material, presumptively lentil, is rare but may represent an overlooked cause of sudden death. In the absence of obvious airway obstruction, diagnosis depends on careful histopathological examination, including recognition of characteristic seed storage cell morphology, including lobulated outlines, optically empty centers, and a PAS-positive outer coating, and correlation with the surrounding acute inflammatory response and foamy macrophage reaction to confirm antemortem aspiration. This is especially relevant in medicolegal practice, where classic predisposing factors such as advanced age or neurological impairment may be absent, and aspiration may not be the initial suspicion. Heightened forensic awareness, meticulous airway examination, and targeted histopathological sampling can improve detection and reporting of these potentially preventable deaths.
